# Vitamin D deficiency, endothelial function and bone biomarkers in post-kidney transplantation patients from North India

**DOI:** 10.1007/s11255-018-2014-7

**Published:** 2018-11-19

**Authors:** Ashok Kumar Yadav, Sanveer Tiwana, Matthew Steel, Raja Ramachandran, Juan C. Kaski, Vivekanand Jha, Debasish Banerjee

**Affiliations:** 1 Department of Nephrology, Post Graduate Medical Education and Research, Chandigarh, India; 2 Cardiology Clinical Academic Group, Molecular and Clinical Sciences Research Institute, St Georges University of London, London, UK; 3 George Institute for Global Health, New Delhi, India; 4 Renal and Transplantation Unit, St Georges University Hospital NHS Foundation Trust, London, UK; 5 Renal and Transplantation Unit, St Georges University Hospital NHS Foundation Trust, Cardiology Clinical Academic Group, Molecular and Clinical Sciences Research Institute, St Georges University of London, Second Floor, Grosvenor Wing, Blackshaw Road, Tooting, London, SW19 8TN UK

**Keywords:** Kidney transplantation, Endothelial dysfunction, Fibroblast growth factor 23, Vitamin D, Cardiovascular

## Abstract

**Purpose:**

CKD patients after kidney transplantation continue to suffer from elevated CV events which may be related to low vitamin D and its adverse impact on vascular function. The prevalence of vitamin D deficiency in North Indian kidney transplantation patients and its impact on vascular and bone biomarkers is unknown which this study investigated.

**Methods:**

Non-diabetic, stable, > 6 months post-kidney transplantation patients, not on vitamin D supplementation, were recruited after informed consent. Data on demographics, anthropometrics and treatment were collected. Blood samples were stored at − 80 °C until analysis for bone and endothelial cell biomarkers using standard ELISA techniques.

**Results:**

The clinical characteristics were: age 37.4 ± 9.9 years, 80% men, 27% ex-smokers, BP 125.5 ± 15.7/78.6 ± 9.7 mmHg, cholesterol 172.0 ± 47.8 mg/dL, hemoglobin 12.6 ± 2.3 g/dL, calcium 9.5 ± 0.6 mg/d and iPTH 58.4 ± 32.9 ng/mL and vitamin D 36.5 ± 39.8 nmol/L. Patients with vitamin D < 37.5 nmol/L (66%) had similar age, serum creatinine, serum phosphate, iPTH, blood pressure but lower calcium (9.3 ± 0.7 vs. 9.6 ± 0.5 mg/dL; *p* = 0.024), lower FGF23 (median 18.8 vs. 80.0 pg/mL; *p* = 0.013) and higher E-selectin (15.8 ± 7.9 vs. 13.0 ± 5.5 ng/mL; *p* = 0.047). On Univariate analysis, E-selectin (*r* = − 0.292; *p* = 0.005), FGF23 (*r* = 0.217; *p* = 0.036) and calcium (*r* = 0.238; *p* = 0.022) were significantly correlated with vitamin D levels. On stepwise multiple regression analysis, only E-selectin was associated with vitamin D levels (*β* = − 0.324; *p* = 0.002).

**Conclusion:**

Vitamin D deficiency was common in kidney transplant recipients in North India, associated with low FGF23 and high E-selectin. These findings suggest further investigations to assess the role of vitamin D deficiency-associated endothelial dysfunction, its implications and reversibility in kidney transplantation recipients.

## Background

The high mortality due to cardiovascular events in chronic kidney disease (CKD) patients is not fully explained by traditional risk factors, leading to exploration of the contribution of non-traditional risk factors such as vitamin D deficiency. Vitamin D deficiency has been associated with endothelial dysfunction [1] and atherosclerosis in general population and in subjects with CKD [2, 3]. Cardiovascular events are the commonest cause of mortality post-kidney transplantation, fifty-fold higher than general population [4].

Fibroblast growth factor (FGF) 23, a phosphaturic hormone, is affected by vitamin D and associated with CV risk in CKD [5, 6]. FGF23 reduces vitamin D synthesis and increases serum PTH [7]. Serum FGF23 level is increased in hemodialysis patients and is associated with mortality [8]. High FGF23 is also associated with cardiovascular mortality in CKD patients [9]. Some recent studies have reported strong association of FGF23 with endothelial dysfunction and cardiovascular risk in CKD subjects [6, 10]. FGF23 declines post-kidney transplantation, but the relationship with vitamin D levels has not been investigated [11].

The relationship of vitamin D deficiency with endothelial dysfunction has been investigated in Indian CKD patients but not in kidney transplantation recipients, who are relatively younger and recipient of live donor kidneys. We have previously demonstrated poor endothelial function in post-transplant and CKD subjects [12] which improves with vitamin D supplementation in CKD patients [13, 14]. In this study, we investigated the relationship of vitamin D deficiency with FGF23 and biomarkers of endothelial dysfunction (E-selectin) in North Indian kidney transplant patients.

## Materials and methods

This cross-sectional study was carried out between December 2013 and December 2014 at the Postgraduate Institute of Medical Education and Research, Chandigarh, India. Adult (18–70 years), clinically stable kidney transplant recipients who had received their grafts at least 6 months ago were enrolled. Patents with abnormal renal function (serum creatinine > 1.5 mg/dL), history of hospitalization in last 6 months, those on any vitamin D supplementation, history of rejection, recurrent nephrotic syndrome, evidence of chronic diseases like diabetes mellitus, heart failure, liver disease, recent infections and significant proteinuria were excluded. All clinical and demographic parameters were recorded for each patient.

Venous blood sample was collected in serum separating tube, centrifuged at 2000 rpm for 25 min and separated serum stored at − 80 °C for biomarker analysis.

### Biomarker assays

Serum samples were used in duplicates to analyze all biomarkers. Total 25 (OH) vitamin D concentration was measured using a commercially available enzyme immunoassay (EIA) kit (IDS Laboratory, UK). The sensitivity of this assay was 5 nmol/L. The intra- and inter-assay coefficients of variation of this assay were 6.7% and 8.7%, respectively. FGF23 concentrations were measured by a commercial ELISA kit (Immutopics, Inc., San Clemente, CA, USA). The sensitivity of the assay was 1.0 pg/mL, intra-assay coefficient of variation was 2.6–4.4%, and inter-assay coefficient of variation was 6.1–6.5%. E-selectin was measured using ELISA (Invitrogen, Frederick, MD). The limit of detection of E-selectin was determined to be less than 0.33 ng/mL (mean of 6 independent assays). The intra-assay and inter-assay coefficient of variation is 5.4% and 6.0%, respectively. Intact PTH was measured using Ray Bio EIA Kit (Ray Biotech, Inc. Norcross, GA, USA). All samples were diluted 4 times before the assay. The minimum detectable concentration was 1.27 pg/mL. The intra- and inter-assay coefficients of variation of this assay were < 10% and < 15%, respectively. Serum calcium and phosphorus were measured on the Cobas C111 automated analyzer (Roche Diagnostics, Indianapolis, IN, USA).

### Statistical analysis

Data are presented as mean ± SD or median and interquartile range. All data were tested for normality of distribution. Continuous variables were compared with independent samples Student’s* t* test if normally distributed, or with Mann–Whitney* U* test. Correlations were tested with Spearman’s rank correlation coefficient. Stepwise multiple regression analysis was performed to test independent association of vitamin D with other parameters. Two-tailed *p* value < 0.05 was considered as statistically significant. All analyses were conducted using the SPSS software version 24.0 (IBM).

## Results

A total of 93 patients were enrolled. The mean age of the patients was 37.4 ± 9.9 years. The study included 18 (19.4%) female and 75 (80.6%) male patients. The cause of end stage renal disease was chronic glomerulonephritis, chronic interstitial nephritis and autosomal dominant polycystic kidney disease in 44 (47.3%), 7 (7.5%) and 5 (5.4%), respectively, and the basic disease was not known in 37 (39.8%). Twenty-seven percentage of the study participants were ex-smokers. The mean body mass index was 20.8 ± 3.3 kg/m^2^. The mean serum creatinine was 1.3 ± 0.3 mg/dL. The mean total cholesterol was 172.0 ± 47.8 mg/dL (Table 1).

**Table 1:** Demographic, detailed clinical and biochemical characteristic and serum biomarkers in study subjects

Parameters	*N* = 93
Age (years)	37.4 ± 9.9
Male/female	75/18
Ex-smoker	25 (27%)
Body mass index (kg/m^2^)	20.8 ± 3.3
Systolic blood pressure (mmHg)	125.3 ± 15.2
Diastolic blood pressure (mmHg)	78.6 ± 9.7
Creatinine (mg/dL)	1.3 ± 0.3
Hemoglobin (mg/dL)	12.6 ± 2.3
Urea (mg/dL)	39.5 ± 15.3
Fasting blood sugar (mg/dL)	90.4 ± 17.4
Total cholesterol (mg/dL)	172.0 ± 47.8
Calcium (mg/dL)	9.5 ± 0.6
Inorganic phosphorus (mg/dL)	3.3 ± 0.8
25 (OH) vitamin D (nmol/L)	36.5 ± 39.8
E-selectin (ng/mL)	14.8 ± 7.3
FGF23 (pg/mL)	75.3 ± 85.3
iPTH (pg/mL)	58.4 ± 32.9

Data presented as mean ± standard deviation and number (percentage)

*FGF23* fibroblast growth factor 23, *iPTH* intact parathyroid hormone

**Fig. 1:**
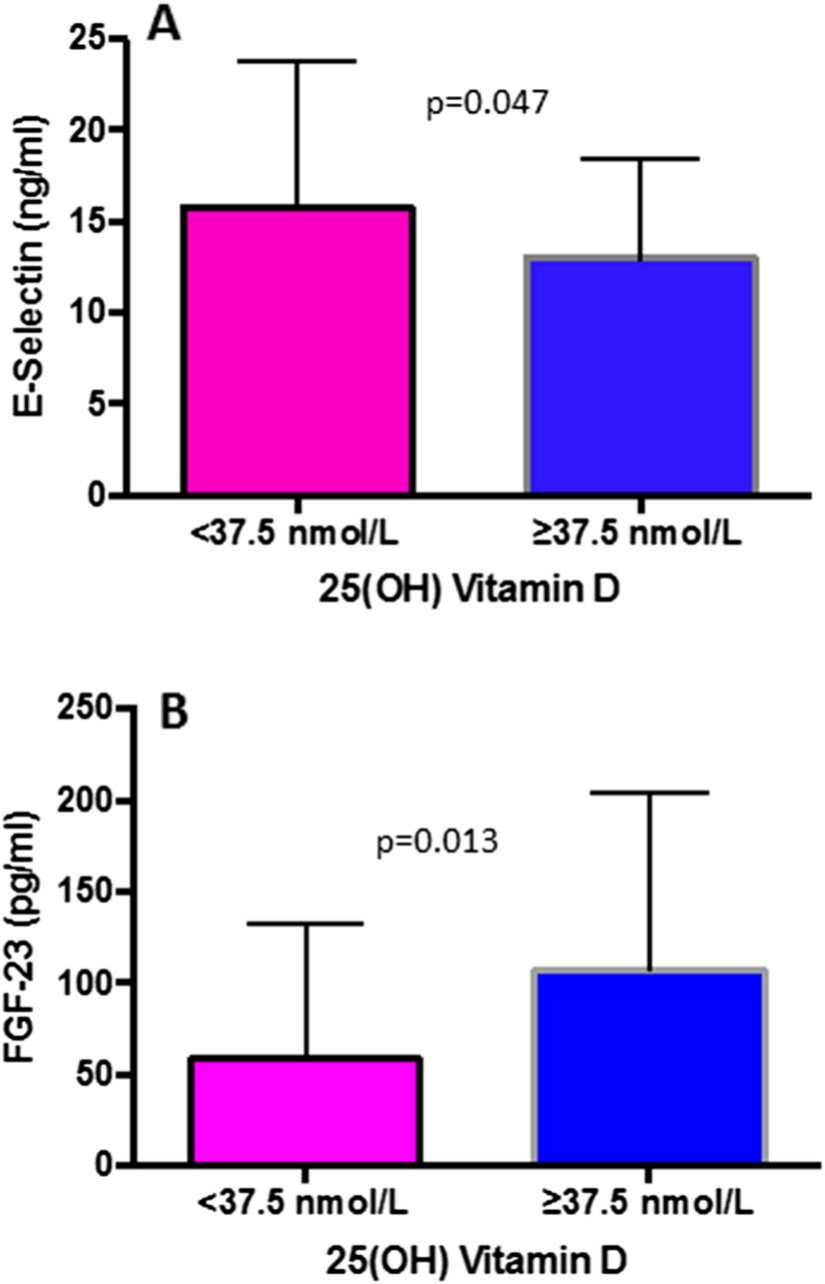
Bar graph showing the serum level of E-selectin and FGF23 in renal transplant recipients with vitamin D level < 37.5 nmol/L and ≥ 37.5 nmol/L. **a** E-selectin was significantly low, and **b** FGF-23 was high in renal transplant recipients with vitamin D ≥ 37.5 nmol/L as compared to vitamin D level < 37.5 nmol/L

The mean serum calcium and phosphorus were 9.5 ± 0.6 and 3.3 ± 0.8 mg/dL, respectively. The mean serum 25 (OH) vitamin D was 36.5 ± 39.8 nmol/L. The mean FGF23, iPTH and E-selectin were 75.3 ± 85.3 pg/mL, 58.4 ± 32.9 pg/mL and 14.8 ± 7.3 ng/mL, respectively. On comparing 25 (OH) vitamin D deficient (< 37.5 nmol/L) and non-deficient (≥ 37.5 nmol/L) patients, those with 25 OH vitamin D deficiency had higher E-selectin (*p* = 0.047) and lower FGF23 (*p* = 0.013) compared to non-deficient patients (Table 2; Fig. 1a, b). There were no significant differences in the other parameters (Table 2). On univariate analysis, E-selectin (*r* = − 0.292, *p* = 0.005), FGF23 (*r* = 0.217, *p* = 0.036) and calcium (*r* = 0.238, *p* = 0.022) significantly correlated with vitamin D levels. On stepwise multiple regression analysis, only E-selectin was associated with vitamin D levels (*β* = − 0.324, *p* = 0.002).

**Table 2:** Patient characteristic according to vitamin D category

	25(OH) Vit D ≥ 37.5 nmol/L (*N* = 32)	25(OH) Vit D < 37.5 nmol/L (*N* = 61)	*p* value
Age (years)	38.5 ± 12.2	36.9 ± 8.5	0.504
Body mass index (kg/m^2^)	20.8 ± 3.0	20.9 ± 3.5	0.888
Systolic blood pressure (mmHg)	123.1 ± 14.8	126.5 ± 15.3	0.301
Diastolic blood pressure (mmHg)	78.3 ± 9.8	78.8 ± 9.6	0.808
Hemoglobin (mg/dL)	12.4 ± 2.2	12.8 ± 2.3	0.515
Serum creatinine	1.3 ± 0.3	1.2 ± 0.4	0.165
Serum urea (mg/dL)	39.7 ± 12.0	39.4 ± 17.1	0.941
Total cholesterol (mg/dL)	180.0 ± 57.6	168.1 ± 42.6	0.465
Calcium (mg/dL)	9.6 ± 0.5	9.3 ± 0.7	0.024
Inorganic phosphorus (mg/dL)	3.3 ± 0.57	3.3 ± 0.93	0.937
25 (OH)D (nmol/L)	53.1 ± 11.7	22.4 ± 7.2	< 0.0001
E-selectin (ng/mL)	13.0 ± 5.5	15.8 ± 7.9	0.047
FGF23 (pg/mL)*	80.0 (17.8–185.4)	18.8 (17.4–95.6)	0.013
iPTH (pg/mL)	53.3 ± 35.0	61.1 ± 31.8	0.294

Data presented as mean ± standard deviation except for *FGF23 which is presented as median (25th–75th percentile)

*FGF23* fibroblast growth factor 23, *iPTH* intact parathyroid hormone

Patients with E-selectin levels in their third tertile had lower 25-OH vitamin D compared to those in the first tertile. There was no difference in FGF-23, iPTH and other clinical/ biochemical parameters between various tertiles (Table 3).

**Table 3:** Patient characteristic according to E-selectin tertiles

	Tertile 1	Tertile 2	Tertile 3
Age (years)	38.6 ± 10.7	36.9 ± 10.4	36.8 ± 8.7
Body mass index (kg/m^2^)	20.4 ± 3.6	19.9 ± 3.0^a^	22.1 ± 2.9^b^
Hemoglobin (mg/dL)	12.6 ± 2.3	12.1 ± 2.4	13.2 ± 1.9
Serum creatinine	1.3 ± 0.4	1.3 ± 0.3	1.2 ± 0.4
Serum urea (mg/dL)	37.9 ± 14.1	42.1 ± 15.0	38.2 ± 17.1
Total cholesterol (mg/dL)	170.0 ± 41.0	178.7 ± 61.8	167.7 ± 41.7
Calcium (mg/dL)	9.5 ± 0.6	9.4 ± 0.7	9.4 ± 0.6
Inorganic phosphorus (mg/dL)	3.3 ± 1.1	3.3 ± 0.6	3.2 ± 0.7
25 (OH)D (nmol/L)	36.6 ± 18.7	34.8 ± 15.9^a^	27.08 ± 15.3^b^
E selectine (ng/mL)	8.0 ± 1.7	13.2 ± 2.0	23.2 ± 5.8
FGF23 (pg/mL)*	72.8 (17.7–145.5)	19.3 (17.7–116.3)	19.5 (17.0–94.8)
iPTH (pg/mL)	61.4 ± 33.8	61.7 ± 33.4	52.3 ± 31.8

Data presented as mean ± SD except for *FGF23 which is presented as median (25th–75th percentile)

*FGF23* fibroblast growth factor 23, *iPTH* intact parathyroid hormone

^a^*p* value < 0.05, compared to tertile 3

^b^*p* value < 0.05. compared to tertile 1

## Discussion

In the present cross-sectional study, we correlated marker of endothelial dysfunction (E-selectin), FGF23 and iPTH with serum 25 (OH) vitamin D levels in renal transplant recipients with stable graft function. Salient findings in the study were increased levels of E-selectin and lower FGF 23 in patients with vitamin D deficiency.

Renal dysfunction is associated with endothelial dysfunction, which is a cause of premature atherosclerosis in the patients with CKD [1, 15]. Renal transplantation is the best treatment for end stage renal disease; however, even successful renal transplantation does not reverse all cardiovascular dysfunction. Low levels of vitamin D are associated with major cardiovascular events, e.g., myocardial infraction, stroke and mortality related to cardiovascular and cerebrovascular diseases [16–19]. Multiple cross-sectional studies have reported association of vitamin D with endothelial dysfunction [1, 20]. Yildirim et al. [21] evaluated endothelial dysfunction in 109 renal transplant recipients, all the patients underwent flow mediated dilatation (FMD), serum 25-OH vitamin D and FGF 23 level. Vitamin D and FGF-23 levels were compared between patients with normal and abnormal endothelial functions. Seventy-two percent of the transplant recipients had endothelial dysfunction, and 80% had vitamin D deficiency. Patients with abnormal endothelial function had lower vitamin D levels compared to patients with normal endothelial function. FGF23 level was numerically higher in patients with abnormal endothelial compared to those with normal endothelial function, but this difference did not reach statistical significance. On a stepwise multivariate regression analysis, only 25(OH) vitamin D independently predicted endothelial dysfunction. Malyszko et al. [22] evaluated the relation of FGF23 and Klotho in 84 stable renal transplant recipients with endothelial function markers; von willebrand factor (vWF), intercellular adhesion molecule (ICAM), vascular cell adhesion molecule (VCAM) and interleukin-6 (IL-6). The authors observed higher levels of FGF-23 and lower Klotho levels in renal transplant recipients compared to healthy controls. FGF23 correlated with copeptin, IL-6, VCAM, platelet count, time after transplantation, vWF and serum calcium levels. The observation let to conclusion of disturbed FGF23-Klotho system in patients with endothelial injury. Higher levels of E-selectin in patients with vitamin D deficiency observed in the present study suggest significant association of endothelial dysfunction with vitamin D levels and are similar to that reported by Yildirim et al. [21].

In our study, we found the significant inverse association of E-selectin with 25(OH) D level which remained significant in multiple regression analysis. This indicates that 25(OH) D may predict endothelial dysfunction in renal transplant patients. This study is in agreement with the study of Yildrim et al. [21] which show the endothelial dysfunction in subjects with low 25(OH)D. We did not find any association of FGF23 with endothelial function which, again, was in agreement with the study of Yildrim et al. [21]. No study has previously demonstrated a relationship of FGF 23 with endothelial dysfunction in kidney transplantation patients. However, such relation is known in general population and CKD patients [10, 23]. In a recent study, FGF23 was independently associated with cardiovascular mortality post-renal transplantation and it remained significant even after adjustment for markers of mineral metabolism and other cardiovascular risk factors, emphasising the possible role of FGF-23 [24]. A case-control study in general population by Tarcin et al. [25] reported that subjects with vitamin D deficiency had a lower FMD as compared to the subjects with sufficient vitamin D levels, and London et al. [20] showed a correlation between 25(OH)D levels and FMD in patients on dialysis. Observational studies indicate that vitamin D is an independent predictor of endothelial function (FMD) in non-dialysis CKD patients; all suggest an active impact of vitamin D on vascular function in non-transplant CKD and general population [1, 26].

The limitations of the study are its small sample size and cross-sectional design. The study shows only relationship of 25(OH)D, FGF23 and E-selectin and does not provide cause and association analysis. However, it is the first study in North Indian kidney transplantation population which evaluates vitamin D deficiency, biomarker of endothelial function and FGF23.

In conclusion, this study shows that vitamin D deficiency is associated with markers endothelial dysfunction and increased FGF23 in renal transplant subjects. Further, studies are required to confirm the association in a larger cohort and clinical trials to investigate whether supplementation of vitamin D improves these parameters in renal transplant patients.
